# Mechanisms and mediation in survival analysis: towards an integrated analytical framework

**DOI:** 10.1186/s12874-016-0130-6

**Published:** 2016-02-29

**Authors:** Jonathan Pratschke, Trutz Haase, Harry Comber, Linda Sharp, Marianna de Camargo Cancela, Howard Johnson

**Affiliations:** Department of Economics and Statistics, University of Salerno, Via Giovanni Paolo II, 132, Fisciano, 84084 Italy; Social & Economic Consultant, Templeogue Road, Terenure, Dublin, 6W Ireland; National Cancer Registry Ireland, Building 6800, Cork Airport Business Park, Kinsale Road, Cork, Ireland; Institute of Health & Society, Newcastle University, The Baddiley-Clark Building, Richardson Road, Newcastle upon Tyne, NE2 4AX UK; Health Intelligence Unit, Health Service Executive, Red Brick House, Stewarts Hospital Campus, Palmerstown, Dublin, 20 Ireland

**Keywords:** Causal modelling, Mediation analysis, Social inequalities, Discrete-time survival model, Structural equation modelling, Deprivation index, Ireland, Colon cancer

## Abstract

**Background:**

A wide-ranging debate has taken place in recent years on mediation analysis and causal modelling, raising profound theoretical, philosophical and methodological questions. The authors build on the results of these discussions to work towards an integrated approach to the analysis of research questions that situate survival outcomes in relation to complex causal pathways with multiple mediators. The background to this contribution is the increasingly urgent need for policy-relevant research on the nature of inequalities in health and healthcare.

**Methods:**

The authors begin by summarising debates on causal inference, mediated effects and statistical models, showing that these three strands of research have powerful synergies. They review a range of approaches which seek to extend existing survival models to obtain valid estimates of mediation effects. They then argue for an alternative strategy, which involves integrating survival outcomes within Structural Equation Models via the discrete-time survival model. This approach can provide an integrated framework for studying mediation effects in relation to survival outcomes, an issue of great relevance in applied health research. The authors provide an example of how these techniques can be used to explore whether the social class position of patients has a significant indirect effect on the hazard of death from colon cancer.

**Results:**

The results suggest that the indirect effects of social class on survival are substantial and negative (-0.23 overall). In addition to the substantial direct effect of this variable (-0.60), its indirect effects account for more than one quarter of the total effect. The two main pathways for this indirect effect, via emergency admission (-0.12), on the one hand, and hospital caseload, on the other, (-0.10) are of similar size.

**Conclusions:**

The discrete-time survival model provides an attractive way of integrating time-to-event data within the field of Structural Equation Modelling. The authors demonstrate the efficacy of this approach in identifying complex causal pathways that mediate the effects of a socio-economic baseline covariate on the hazard of death from colon cancer. The results show that this approach has the potential to shed light on a class of research questions which is of particular relevance in health research.

## Background

A wide-ranging debate has taken place in recent years on mediation analysis and causal modelling [1–9]. This debate has involved many different fields and raised profound questions about the status of scientific explanations, statistical theory and research methodology when making causal inferences. In this paper, we build on this discussion to outline an integrated approach to the analysis of research questions that situate survival outcomes in relation to complex causal pathways. There are good reasons for pursuing this goal, as researchers are increasingly seeking to shed light on the “mechanisms” that generate survival outcomes by exploring mediated effects. As Aalen et al*.* [1] observe, “In other areas [outside Psychology and Social Science] mediation analysis has largely been ignored. This is especially so for situations where time plays a central role, as in survival analysis. In view of the importance of survival analysis in medicine and other areas, it is surprising that not more attention has gone into the issue of mediation.”

The background to this contribution is the increasingly urgent need for policy-relevant research on the nature and form of social inequalities in relation to health and health care, as interventions to promote population health and to improve equity rest on causal interpretations of the determinants of health-related outcomes, however incomplete or flawed these may be [10]. At the same time, and despite the enormous progress that has been made in each of the aforementioned areas, an integrated framework for causal modelling has not yet been identified in health research, with a view to incorporating survival outcomes with such desirable features as (a) latent variables, (b) time-varying covariates, (c) complex pathways and (d) support for causal inferences in relation to direct and indirect effects.

We will begin by briefly summarising recent debates on causal inference, mediated effects and statistical models. We will show that these three strands of research have powerful synergies which can be exploited by bringing them together within an appropriate analytical framework. We will then present an illustrative example using survival data for a sample of patients diagnosed with colon cancer in the Republic of Ireland between 2004 and 2008. We will assess whether social class (measured by a proxy variable) exerts statistically-significant direct and indirect causal effects on survival prospects. We are particularly interested in assessing whether the influence of this socio-economic baseline covariate is mediated by the route of admission to or by the caseload of the hospital where the main treatment was received. These indirect pathways are of great relevance from a policy-making perspective, as they have the potential to shed light on the mechanisms that (re)produce social inequalities in health outcomes.

### Literature review

#### Mediation effects

The study of mediation raises complex issues, although the basic structure of such effects is simple. By including mediators in a regression equation, the coefficients for other variables in the model may change or become statistically or substantively non-significant. In this way, mediation effects can mask the influence of certain variables and impede a full appreciation of their role in determining outcomes. Conversely, the appropriate specification of such effects can provide practitioners and policy-makers with richer information on disparities in access to health and health care.

Mediation analysis has stimulated interest amongst health researchers due to its potential to provide answers to a series of important research questions and due to dissatisfaction with the methods and approaches which have tended to dominate health research [4]. The latter have recently been called into question, primarily due to their tendency to focus on empirical associations (“black-box epidemiology”) and consequent failure to develop plausible explanations [3, 11]. As a possible solution to this problem, “mechanisms” have been contrasted with “black boxes”. The aim of applied research, it is argued, should be to develop increasingly sophisticated accounts of the systemic relationships and processes that generate empirical regularities [12]. In this vein, mediation analysis can inform intervention strategies, identify “active ingredients” and suggest strategic sites for action.

Where the mediator and outcome are singular, continuous and observed, multiple-equation techniques for studying mediation are frequently used, building on Baron and Kenny’s influential approach [13, 14]. As these have been widely discussed, we will merely note that this technique relies on a series of linear regression models and enables the researcher to assess whether a single variable may be said to mediate between a covariate and the outcome [15]. Although these techniques have been applied countless times, they are of limited use if either the mediator or the outcome are categorical or ordinal (or represent the time to an event), or if more complex forms of mediation are involved [5, 8]. These limitations have discouraged health researchers from exploring mediation effects, partly due to the fact that non-linear models like the Cox model make it difficult to estimate indirect effects [16].

#### Causal inference

Causality has become a major issue in Statistics in recent years [1]. The “traditional” statistical approach to the analysis of direct effects involved conditioning on a mediating variable. Aware that this does not rest on a rigorous definition of causality, Robins and Greenland [17] and Pearl [18] developed alternative formulations. The “causal inference” literature which subsequently developed relies on the counterfactual theory of causality proposed by Rubin [19]. Judea Pearl, an influential scholar in this area, contributed to the new-found popularity of causal questions amongst statisticians by combining Rubin’s approach with the theory of non-parametric Structural Equation Models. Other authors have used similar techniques to clarify the necessary and sufficient conditions for making causal inferences about mediation effects [6, 20].

Within this literature, causal inference focuses on four different kinds of effects: the total effect, the “controlled” direct effect (based on the idea of holding the mediating variables fixed by setting their values to a constant by some kind of intervention), the “natural” direct effect (where the treatment is set at a given level and we compare outcomes without fixing the mediators to a constant, but allowing them to assume the “natural” levels that they would have taken in the absence of the treatment) and the “natural” indirect effect (where the direct effect is disabled and we focus on the effect transmitted by the mediator).

Pearl, in a recent paper [10], clarified some of the issues at stake when making causal inferences about mediation effects using statistical models. Firstly, he argues that indirect effects should not be treated as artefacts or nuisance parameters, but as “an intrinsic property of reality that has tangible policy implications”. The second is that it is possible to define direct and indirect effects within a general, causal approach that does not require particular distributional assumptions. Thirdly, he shows that the assumptions required by causal mediation analysis are essentially analogous to those that apply to causal models more generally: no confounding due to unmeasured common causes. Fourthly, he demonstrates that the total effect, natural direct effect and natural indirect effect are identified for linear Structural Equation Models as long as the aforementioned assumptions are satisfied and can be estimated in a straightforward way from the estimated coefficients. Finally, Pearl considers such models to be potentially useful despite their reliance on assumptions which cannot be tested explicitly.

This raises interesting questions about the relationship between statistical models, generative mechanisms and causality – which hinge around a fundamental paradox. Although statistical models can permit valid inferences about causal mechanisms under certain conditions, the very nature of these models implies that these conditions will rarely, if ever, be (fully) satisfied. After all, reality is infinitely complex, whilst models provide relatively simple, stylised representations, and researchers can never be certain that they have included all relevant confounders.

One way of tackling this paradox is to embed it within the process of scientific discovery. The plausibility of models is assessed by the scientific community using prevailing criteria and techniques, which either reinforces or undermines the conviction that a model captures the essence of a really-existing mechanism. If a model omits an important confounder, the onus is on other researchers to demonstrate that alternative specifications yield different conclusions. In other words, it is not sufficient to appeal to the *possibility* of misspecification or omission (which applies to all models); this must be substantiated explicitly.

The impact of model misspecification depends on the strength of the effects associated with the omitted variables or paths, which implies that once substantively-important covariates have been included in a model, the omission of less important effects will, *ceteris paribus*, have a weaker influence on the model. Rather than seeking a warrant for making absolute claims, we would suggest that the aim of causal models is to clarify important relationships and pathways and to contribute to the development of mechanism-based explanations.

#### Statistical models for mediation analysis

In an attempt to overcome the limitations of existing approaches to mediation analysis, researchers have sought to extend the Baron-Kenny approach to survival outcomes by applying them directly to Cox models [21, 22]. This technique is known to yield biased results, however, and has met with forceful criticism in the scientific literature, as summarised by Lange and Hansen [23]:

*Most importantly, the observed changes in hazard ratios cannot be given a causal interpretation. In addition, the important assumption of proportional hazards can never be satisfied for both models with and without the mediator. In other words, it is not mathematically consistent to use a Cox model both with and without a potential mediator (mathematically, this is due to the fact that the class of proportional hazard models is not closed under marginalization).*

As a result of these difficulties, researchers have concentrated their efforts on extending survival models in different ways. One such approach uses “marginal” models and focuses on obtaining causally-valid inferences for single mediation effects using standard survival models [24]. Another approach – known as “marginal structural modelling” - can be used to identify the causal effect of time-dependent exposures while controlling for time-dependent confounders which are also affected by the exposure [25]. These models use inverse probability of treatment weights and inverse probability of censoring weights to create a pseudo-population in which treatment is un-confounded by subject-specific characteristics or censoring [26]. The models are therefore designed to remove confounding due to a specific type of mediation effect, rather than to study mediation effects more generally. The independent variable of interest has to be dichotomous and their integration with survival outcomes is limited.

The third approach uses Dynamic Path Analysis, developed by Fosen et al*.* [27] using Aalen’s additive hazards model, as “an extension of classical path analysis to a time-continuous survival setting where path effects are estimated as a function of time” [16]. Lange and Hansen [23] suggest that this approach has weaknesses when used to study mediation, as it cannot sustain causal interpretations and cannot be implemented using standard software. Their recommendation is to adapt the additive hazards model in a different way to calculate the counterfactual rate difference, which represents the number of deaths that can be attributed to mediation through the mediator, compared with those that can be attributed to the direct path. Martinussen and Vansteelandt [28] also use the Aalen additive hazards model to adjust survival models for confounding in a similar way.

These approaches *seek to extend existing survival models* to obtain valid estimates of causal effects. As a consequence, they encounter constraints on the number and kinds of variables that can be analysed, and more complex causal mechanisms typically cannot be assessed. An alternative strategy is to integrate survival outcomes within Structural Equation Models, as the latter already include specifications such as growth curves, multilevel structures, latent variables, latent classes and multiple outcome variables [29]. Iacobucci [5] offers a general motivation for this strategy:

*Mediation models have also been generalized to allow for nomological networks that are richer than just the three central constructs, X, M, and Y. If there are additional predictors or consequences of any of these, Structural Equation Models are superior (i.e., mathematically statistically optimal given their smaller standard errors), substantively to get a better sense of the bigger theoretical picture, and statistically because the focal associations will be estimated more purely, having other effects partialed out and statistically controlled…*

We favour this strategy, which seeks to integrate survival outcomes within a Structural Equation Model, not least because the latter has come to be seen as the most appropriate methodological framework for carrying out mediation analysis more generally [10, 30–33]. The nature of survival models has, for a long time, appeared to exclude this possibility [5]. We will show in the next section how this challenge may be tackled, preparing the ground for an integrated framework.

#### Structural equation modelling

There is an intuitively appealing way of integrating time-to-event data within Structural Equation Models. The idea of using a linear specification of the hazard function based on discrete-time modelling techniques was proposed more than 20 years ago, and Singer and Willett [34] showed that this model could be estimated using the tools of traditional logistic regression analysis. Muthén and colleagues subsequently integrated the discrete-time survival model within the MPlus program [35, 36]. This approach – which will be described in greater detail below – makes it possible to estimate complex discrete-time survival models using existing software. It is possible, for example, to relate survival outcomes to other kinds of data structures and to develop models which more accurately reflect real-world mechanisms:

*Discrete-time models have the strength that they can easily accommodate time-varying covariates. They also do not require a hazard-related proportionality assumption that is commonly used in continuous-time survival analysis, for example, the Cox proportional hazards model. In addition, these models easily allow for unstructured as well as structured estimation of the hazard function at each discrete time point.* [35]

This conceptual shift – from continuous to discrete time, and from a single equation to a Structural Equation Model – permits the kind of integration of methods that is required for mediation analysis to yield its full potential in health research. Amongst the benefits of this approach are that it encourages researchers to formulate and test more comprehensive hypotheses and to develop more ambitious theories regarding generative mechanisms.

The notion of developing and testing *mechanism-based* accounts of the world involves a metaphorical mapping which is highly effective in this context. One way of understanding this concept is to situate it, once again, within the process of scientific discovery, whereby a little-understood association may be replaced, over time, by a more detailed explanation. This process gives rise to a constant revision of explanations, accompanied by new and more powerful accounts which articulate the relationship between processes situated at different levels. We argue that the central aim of scientific research is to provide an increasingly accurate or powerful account of these “mechanisms”.

The mechanism-based approach can be applied effectively to the development of statistical models. Models offer a stylised representation of real-world mechanisms; by interpreting the results of statistical models, we can make substantiated claims about the ways in which these mechanisms work. In fact, “direct” and “indirect” effects always relate to a specific theory/model, as “typically, there are other (unmeasured) intermediate variables that would mediate the direct effect” [3]. Indeed, every direct effect in a statistical model may be treated as a “black box”, and replaced (over time) by a more complex set of direct and indirect effects. It is the substantive focus of each research project that ultimately decides which black boxes should be opened (simultaneously creating new black boxes).

## Methods

### The discrete-time survival model

In discrete time, *h*_*j*_ denotes the probability that an individual experiences a non-repeatable event during time period *j*, given that he or she did not experience it during previous periods:

1 $$ {h}_j=P\left(T=j\Big|T\ge j\right) $$

where *T* is a discrete random variable that indicates the time period in which the event occurs. The most important aspect of the model, which underwrites its elegance, is that by conditioning on successive periods the statistical theory is simplified [37]. As a consequence, the joint density function for the various time intervals (e.g. *T*_1_, *T*_2_, *T*_3_) can always be written as the product of the marginal distribution of *T*_1_, the conditional distribution of *T*_2_ given *T*_1_ and the conditional distribution of *T*_3_ given *T*_1_ and *T*_2_. As in other survival models, the survival probability, which expresses the probability of *not* experiencing the event, can be expressed in terms of the hazard:

2 $$ {S}_j=\prod_{k=1}^j\left(1-{h}_k\right) $$

where *h*_*k*_ indicates the hazard probability for each time period up to and including *j*, when the event was observed.

A log-odds relationship is often specified between the individual hazards and the covariates [34, 35]. If we assume that **z**_*ij*_ is a *p* × 1 vector of values for a set of time-varying covariates (*z*_1_,…, *z*_*p*_), measured for individual *i* in time period *j* and that **x**_*i*_ is a *q* × 1 vector of values for a set of time-invariant covariates (*x*_1_,…, *x*_*q*_), then the hazard can be related to the covariates using the following logistic function:

$$ {h}_{ij}=\frac{1}{1+{e}^{-\left({\mathrm{logit}}_{ij}\right)}} $$

3 $$ {\mathrm{logit}}_{ij}={\beta}_j+\kappa {\mathit{\hbox{'}}}_{zj}{\mathbf{z}}_{ij}+\kappa {\mathit{\hbox{'}}}_{xj}{\mathbf{x}}_i $$

where *κ*_*zj*_ is a logit parameter vector for the time-varying covariates and *κ*_*xj*_ is a logit parameter vector for the time-invariant covariates, both of which can vary across the *J* time periods [35]. The resulting coefficients can be antilogged and interpreted as odds ratios in the usual way. Both continuous and categorical covariates can be included. If we drop the *j* subscript from *κ*_*zj*_ and/or *κ*_*xj*_, the effects of the covariates are assumed to be equal across time periods, yielding the proportional hazard odds model. The inverse logit of *β*_*j*_ is the hazard probability for time period *j*, where ***z***_*j*_ = 0 and **x** = 0, which gives the *baseline hazard*. A constant baseline hazard probability model can be obtained by setting *β*_*j*_ = *β* for all *j* = 1,…, *J* or, alternatively, a piecewise or parametric baseline hazard function can be specified.

In general terms, therefore, the conditional log-odds that an event will occur in a given time period, given that it did not occur in previous periods, is modelled as a linear function of a constant term (which may or may not be specific to the period) and the values assumed by a set of explanatory variables (which may or may not vary over time), multiplied by a set of appropriate slopes (which may or may not vary across time periods).

To specify the model, we define a *J* × 1 vector **u** of binary variables, for which we imagine a set of underlying continuous latent response propensities, **u**_ij_^*^  = (*u*_*i*1_^*^, *u*_*i*2_^*^, …, *u*_iJ_^*^)’, whereby the latent *u*_ij_^*^ are related to the observed *u*_*ij*_ via a threshold parameter *τ*_*j*_. This is identical to the derivation of logistic regression via the “latent response” formulation. The higher the threshold *τ*, the higher u ^*^ needs to be to exceed it and the lower the probability of *u* = 1. The threshold parameter is related to the intercept by the equation *β*_*j*_ = –*τ*_*j*_.

The binary *u*_*ij*_ = 0 if individual *i* is observed to be at risk for the event of interest for the whole of time period *j* but does not experience it, *u*_*ij*_ = 1 if individual *i* experiences the event in time period *j* and *u*_*ij*_ is missing if individual *i* has already experienced the event or is lost to follow-up (i.e. right-censored). The fact that an individual does not have observations on *u* after experiencing the event or dropping out is handled as missing data, and the conventional assumption of “non-informative censoring” must be made (as in other survival models). The Maximum Likelihood estimator is constructed as a product of terms which coincide with each period up to the last one for which data were recorded, assuming that the *n* individuals composing the sample are independent given the covariates [34, 35].

Expressing the hazard probabilities as a function of the observed covariates using the logit link function is equivalent to the logistic regression of the *u*_*i*_ on the observed covariates [34]. This dependence on the explanatory variables is what introduces heterogeneity and accounts for inter-individual differences in hazard probabilities, yielding a proportional shift in the baseline hazard profile if the coefficients are assumed to be equal (proportional hazards model). The discrete-time survival model with unstructured hazard probabilities but without covariates is always saturated, and thus fits the *u* variables perfectly.

In the MPlus program, the proportional hazards discrete time survival model may be specified either by placing equality constraints on the coefficients for the logistic regression of each *u* variable on each explanatory variable or by creating a latent variable with a variance of 0 and a unit path to each *u*. By relaxing the constraints on these paths, it is possible to test the proportionality assumption. Combinations of discrete-time survival models and other structural equation models (such as latent curve models, for example) can be used in a flexible way to address a wide range of research questions [38]. A final characteristic of the discrete-time survival model that is worth noting is that its estimates converge on those provided by the Cox continuous-time model as the definition of the time periods becomes increasingly fine-grained [39]. The discrete-time model can be justified not only when time of observation is inherently discrete, but also when it is measured continuously and subsequently transformed into discrete intervals. This provides a useful bridge between the two techniques for purposes of comparison.

### Data

We will now provide an illustrative example of the approach outlined above. Our statistical model is based on discrete time-to-event data for death due to cancer of the colon, with time measured in quarters. All cases of adenocarcinoma of colon (ICD10 C18) registered by the Irish National Cancer Registry as incident during the years 2004–2008 are included. This implies a minimum of 13 and a maximum of 32 time intervals, which we truncate at 24, as the number of deaths per quarter is negligible after this point. An unstructured baseline hazard profile is adopted, for simplicity. Registry data were linked to public hospital discharge data from the Hospital Inpatient Enquiry (HIPE) for all patients admitted to public hospitals [40]. Active tumour-directed treatment is defined as excisional biopsy, surgery, chemotherapy or radiotherapy with a primary aim of removing or reducing the tumour.

The type of initial admission (scheduled or emergency) was determined from the HIPE data, and information on patient age and tumour stage (AJCC) was derived from the Registry. Social class is measured by a proxy variable: the small-area affluence/deprivation score of the patient’s neighbourhood of residence using the Haase-Pratschke index of relative affluence and deprivation [41]. Scores on this index are based on 2006 census data (using Small Areas with an average population ~230 persons) and matched to the individual-level data provided by the National Cancer Registry of Ireland by geo-coding patients’ addresses. Treatment is classified as either sub-optimal (less intensive treatment, or fewer modalities than recommended) or optimal/more aggressive (treatment according to guidelines or using additional modalities) by comparison with the recommendations of the National Comprehensive Cancer Network [42].

High caseload for the main hospital was defined as more than 40 colon cancer patients per annum, on average, during the study period. Registry data were also linked to official death certificates from the Central Statistics Office. Deaths were classified as either due to colon cancer or other causes, based on an algorithm developed by the Scottish Cancer Registry [43]. All treatments were recorded for the first 12 months following diagnosis, and patients who received no treatment were excluded from the analysis, as these typically involve cases where cancer was diagnosed either post-mortem or immediately prior to death. Patients were followed until death or censoring at the end of the study (31 December 2011), and those who died from other causes were also treated as censored observations. All explanatory variables included in the model are time-invariant.

Of 6347 colon carcinomas incident in 2004–2008 in patients who did not develop a second primary cancer prior to 31/12/2011, 5178 (81 %) had at least one episode of tumour-directed treatment and 4793 patients (93 %) received cancer-directed surgery. Just over half (55 %) of patients were male and 52 % were aged 70 or over. The majority (60 %) were married and most (63 %) attended hospital solely or predominantly as public patients. Almost half (46 %) of cancers were at stage I or II at diagnosis, and 85 % were of low or intermediate grade. More than three quarters (78 %) of patients had no recorded comorbid conditions and just over one fifth (22 %) were admitted as an emergency. Treatment was classified as optimal (or more aggressive) in 81 % of cases, but only 56 % of patients attending low-caseload hospitals fall into this category.

We coded the survival outcome variables so that patients enter the study at the moment of diagnosis, with staging data providing a proxy for onset of illness (and therefore early/late diagnosis). We include only a small set of baseline covariates (see Table 1 below) to simplify the presentation and due to space considerations; a more fully-specified model will be presented in a separate paper.

**Table 1:** Variables included in the model (*N* = 5178)

*Variable*	*Value*	*Summary data*
Stage at diagnosis (three dummy variables)		
	Stage I (reference)	559 (10.8 %)
	Stage II	1,812 (35.0 %)
	Stage III	1,678 (32.4 %)
	Stage IV	1,129 (21.8 %)
Treatment optimality (binary)^a^		
	sub-optimal (0)	995 (19.2 %)
	optimal/more aggressive (1)	4183 (80.8 %)
Age at diagnosis (continuous measure)	(Scaling factor = 0.10)	
	< 60	1,109 (21.4 %)
	60–69	1,396 (27.0 %)
	70–79	1,779 (34.4 %)
	80+	894 (17.3 %)
Deprivation score (continuous)^b^	(rescaled to 0–1 metric)	
	Mean	0.58
	Standard deviation	0.13
Emergency admission (binary)^c^		
	First admission elective (0)	4,026 (77.8 %)
	First admission via A&E (1)	1152 (22.2 %)
High caseload hospital (binary)^d^		
	Less than or equal to 40 per annum (0)	2,876 (55.5 %)
	More than 40 per annum (1)	2,302 (44.5 %)

^a^Missing values (4.4 %) were assigned to the modal category (optimal or more aggressive)

^b^Missing values (< 1 %) were estimated using the EM algorithm in IBM SPSS Statistics v.20

^c^Missing values (6.5 %) were assigned to the modal category (elective admission)

^d^Missing values (< 1 %) for caseload were replaced using the caseload of hospital where first (rather than main) treatment was received or, if this was not possible, estimated using the EM algorithm in IBM SPSS Statistics v.20

### Model specification

In the example analysis, we use a causal modelling approach to explore whether the social class position of patients has a significant direct and/or indirect effect on the hazard of (cause-specific) death from colon cancer, mediated by route of admission to hospital (elective or emergency) and/or the caseload of the hospital where the main treatment was received. We hypothesise that age and social class influence the route of admission to hospital, as older and more disadvantaged patients are more likely to be admitted as emergency cases. We further hypothesise that access to a high-caseload hospital will depend on age, affluence and route of admission: not only do we expect that older and more disadvantaged patients have a lower probability of accessing high-caseload hospitals, but we also believe that this applies to those who enter hospital on an emergency basis.

**Fig. 1:**
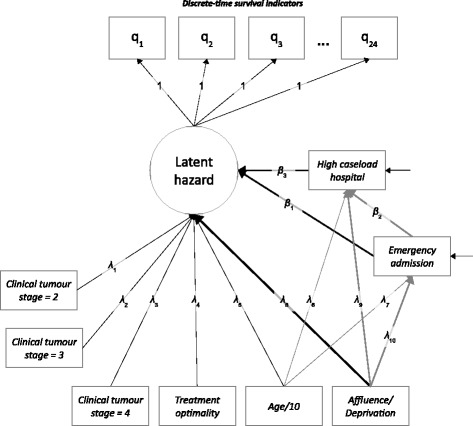
Discrete-time survival model for colon cancer with mediation effects

The causal order encoded by the model is based on logical/theoretical criteria as well as chronological order, and we assume no effect modification. The direct and indirect influences are shown in Fig. 1 below, using the typical conventions of path models, where observed variables are represented by rectangles, latent variables by circles, direct effects by straight arrows which point from cause to effect and residuals by straight arrows pointing at the dependent variable in a regression equation. All covariances are omitted from the diagram, but included for pairs of exogenous variables where direct effects were not specified. The direct and indirect pathways relating to social class are highlighted by thicker arrows in the figure. The upper part of the figure (including the latent hazard and survival indicators) coincides with the model defined in Equation 3, albeit with time-invariant covariates and constant effects (i.e. logit_*ij*_ = *β*_*j*_ + κ’_x_**x**_i_). The latent variable shown in the figure (labelled “Latent hazard”) merely simplifies the presentation, as the direct effect of each explanatory variable on the survival outcome can be identified with a single path. This specification is exactly equivalent to one in which each explanatory variable has an effect on each of the 24 discrete-time survival indicators, with these 24 effects being constrained to be equal.

The coefficients from the logit regression of the survival outcome on the covariates may be interpreted as linear regression coefficients using the threshold approach, as mentioned earlier [44, 45]. It would be attractive to adopt the same procedure for the two mediating variables – admission route and high caseload – which are both binary. Unfortunately, this is not possible in MPlus, which can only handle continuous mediators in models with discrete-time survival outcomes. We therefore use the linear probability model for the equations in which these two mediators are the dependent variables; this procedure is sub-optimal but nevertheless reasonable as the distributions of these variables are relatively balanced [46].

As the entire model is linear (because the discrete-time survival part of the model may be interpreted in terms of a linear regression using the latent response formulation), the indirect effects may be estimated using the product-of-coefficients approach and represent “natural indirect effects”. The direct effects in the statistical model are equivalent to “natural direct effects”, whilst the total effect is given by the sum of the direct and indirect effects [10]. The standard errors for the indirect effects are estimated using the delta method and the model is estimated using MPlus v5.21, with a Maximum Likelihood estimator and robust standard errors [36]. The code used to specify the model is included in Appendix A. The size of the mediation effects is reported below, both in absolute terms and as a mediation proportion, with standard errors and confidence intervals [32]. The latent response variables underlying the survival indicators have a mean of 0 and a standard deviation of 1 and thus the raw coefficients may be interpreted as capturing the effect, measured in standard deviations, of a unit change in the explanatory variables; the units of the latter are shown in Table 1.

## Results

**Fig. 2:**
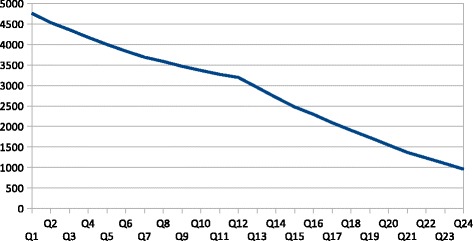
Patients who remain exposed to hazard of death, by quarter from diagnosis

The results of the analysis are shown in Tables 2 and 3 below and the number of patients who were alive in each quarter, from diagnosis, is shown in Fig. 2. As noted above, all variables were assumed to have a constant effect over time, and this assumption is encoded in the unit paths described earlier (Fig. 1), specified between the discrete-time survival indicators and the latent hazard.

**Table 2:** Direct effects on hazard of death, admission route and caseload

Variable	Estimate	S.E.	Est./S.E.	*p*-value
Hazard of death due to colon cancer:				
* Age (/10)*	0.299	0.036	8.208	0.000
* Affluence/deprivation (0*–*1 metric)*	-0.596	0.271	-2.200	0.028
* Emergency admission (binary)*	0.411	0.084	4.886	0.000
* Stage II (compared to Stage I)*	0.639	0.213	2.998	0.003
* Stage III (compared to Stage I)*	1.306	0.210	6.211	0.000
* Stage IV (compared to Stage I)*	3.193	0.207	15.438	0.000
* Treatment optimality (binary)*	-0.766	0.106	-7.234	0.000
* High caseload hospital (binary)*	-0.173	0.071	-2.422	0.015
Admission route via emergency:				
* Intercept*	0.241	0.061	3.968	0.000
* Age (/10)*	0.023	0.007	3.229	0.001
* Affluence/deprivation (0*–*1 metric)*	-0.302	0.062	-4.849	0.000
* Residual variance*	0.170	0.004	38.133	0.000
High caseload hospital:				
* Intercept*	0.192	0.074	2.602	0.009
* Age (/10)*	-0.009	0.008	-1.116	0.264
* Affluence/deprivation (0*–*1 metric)*	0.576	0.077	7.503	0.000
* Emergency admission (binary)*	-0.063	0.023	-2.708	0.007
* Residual variance*	0.241	0.002	131.164	0.000

**Table 3:** Effects of affluence/deprivation on hazard of death

Variable	Estimate	S.E.	Est./S.E.	*p*-value
Direct effect:				
* Affluence/deprivation (0*–*1 metric)*	-0.596	0.271	-2.200	0.028
Indirect effects:				
* 1. affluence → emergency → hazard*	-0.124	0.036	-3.457	0.001
* 2. affluence → caseload → hazard*	-0.099	0.043	-2.296	0.022
* 3. affluence → emergency → caseload → hazard*	-0.003	0.002	-1.689	0.091
* All indirect effects*	-0.227	0.056	-4.056	0.000
Total effect:				
* Affluence/deprivation (0*–*1 metric)*	-0.823	0.27	-3.047	0.002
Mediation proportion:				
* Affluence/deprivation (0*–*1 metric)*	0.276

Starting with the hazard of death due to colon cancer, the model indicates that an increase in age of 10 years leads to an increase in the hazard of 0.30 standard deviations, whilst moving along the spectrum of affluence and deprivation from the most deprived to the most affluent patient leads to a substantial reduction in the hazard (-0.60 standard deviations). Entering hospital for the first time via the emergency department leads to an increase in the hazard of 0.41 standard deviations, and tumour stage has an even greater impact (0.64 for Stage II compared to Stage I, 1.31 for Stage III and 3.19 for Stage IV). Optimal treatment reduces the hazard considerably (-0.77 standard deviations), as does attending a hospital with high caseload for the treatment of colon cancer (-0.17 standard deviations). There is no residual variance and the sample is assumed to be homogeneous (i.e. no “frailty”, no latent classes), in line with standard practice in basic discrete-time survival modelling. All heterogeneity in hazard profiles thus derives from the effects of the explanatory variables, as noted above.

Turning to the admission route, each ten-year increase in age leads to an increase of 0.02 standard deviations in the probability of an emergency admission, whilst the most affluent patients have a lower risk of entering hospital in an emergency when compared with the most deprived (-0.30). As far as hospital caseload is concerned, affluence has a powerful impact (0.58) on the probability of receiving treatment in a high-caseload hospital. Being an emergency case at admission reduces this probability (-0.06), and all effects are statistically significant, with the exception of the effect of age on high caseload.

As can be seen from Table 3, the indirect effects of social class (as measured by affluence/deprivation score) are substantial and negative (-0.23 overall). This implies that, in addition to the substantial direct effect of this variable (-0.60), there are indirect effects that account for more than one quarter of the total effect. Whilst the standard error of the direct effect is relatively large, those of the indirect effects are small. This is because the model has high power to detect indirect effects. The two main indirect effects, via emergency admission (-0.12) and via hospital caseload (-0.10) are of similar size.

## Discussion

The empirical example presented in the previous section demonstrates the flexibility of the causal modelling framework set out earlier and shows its potential in relation to the study of mediation effects. As a result of the way in which causal models bring together theoretical knowledge and empirical evidence, they have the potential to sustain ongoing research programs which yield progressively more refined and powerful explanations. The indirect effects of social class were shown to be substantial in size and statistically significant, accounting for roughly one quarter of the total effect. In a more fully-specified model with a full set of covariates, this proportion is likely to increase.

By improving measurement instruments, including new covariates and modifying the structure of a model such as this, it is possible to provide more appropriate and precise information to practitioners and policy-makers and to sustain an ongoing dialogue regarding mechanisms, possible interventions and monitoring strategies. Rather than merely replicating tests of association between specific variables in an endless series of samples, this approach encourages the progressive enrichment and extension of explanatory models.

The model presented here shows that survival outcomes can be integrated within the framework of causal modelling using the linear specification of the discrete-time survival model. Although the model is simple, it provides valuable additional information compared to alternative approaches to modelling survival. It confirms that there is a risk of underestimating the overall impact of social class on health outcomes when attention is confined to direct effects. As noted earlier, this is because intermediate variables must be included in order to obtain accurate estimates and to assess the influence of treatments and interventions, but their inclusion tends to mask the effects of key baseline covariates. Given the large standard error associated with the direct effect, the influence of social class could easily be overlooked, particularly when working with small samples.

Secondly, the analysis opens up interesting avenues for intervention strategies by providing a better understanding of *how* social class differentials in health outcomes are generated. The model suggests that differences in wealth, knowledge and influence (captured by social class) enable advantaged individuals to seek professional assistance before a problem becomes acute, whilst those who are more disadvantaged encounter greater difficulties in seeking and/or receiving assistance following initial symptoms. As a result, more affluent groups are able to obtain better information about their condition and to decide where to receive treatment, using their resources to choose experienced consultants and to reduce waiting times. What was previously a “black box” is now a potential mechanism, which can be refined and extended in different ways in the course of subsequent research.

This approach set out above has a number of strengths, not least because survival data are themselves frequently based on a discrete conception of time (measured in weeks, months or years). From this perspective, the discrete-time approach offers an intuitively compelling framework that is appropriate to many research problems, although it has rarely been cited by health researchers. For example, the path-breaking paper by Muthén and Masyn [35] has been cited 127 times (Google Scholar, January 2015), mostly by Psychologists, with only two citations in the broad field of medical research.

Many studies suggest that socio-economic variables have a profound influence on health, although the precise pathways through which these effects operate remain unclear [4, 32]. When studying health outcomes, it is often necessary to control for variables such as these. However, once we control for the stage of illness, types of treatment and so on, we may find that these socio-economic measures no longer have significant effects. This is not a problem if we are merely concerned with predicting the outcome, but it could be misleading to base policies on these kinds of findings. It is quite possible, for example, that socio-economic covariates have an influence on intermediate health-related and treatment-related variables, implying that they have *indirect* effects on the outcome. This is a good example of a research problem that requires sophisticated techniques for conducting mediation analysis within an extended nomological network (set of variables and paths).

It is only appropriate to conclude by mentioning some limitations to this analytical framework. Firstly, as we noted above, the statistical theory and software tools for causal mediation analysis with survival outcomes are currently confined to continuous mediators. In our example, we used the linear probability model to regress the binary mediators on the baseline covariates. Secondly, the calculation of indirect effects by the product-of-coefficients method with a survival outcome relies on the latent response formulation (for the regression of the survival indicators on the explanatory variables). Although leading methodologists view this as a valid extension of mediation analysis (see, for example, the responses provided by Linda and Bengt Muthén on the MPlus Discussion Board on December 14 2005, February 09 2010, July 26 2006 and August 18 2008, http://www.statmodel.com/cgi-bin/discus/discus.cgi), a more rigorous statistical justification for this approach would be valuable.

Thirdly, in a fully-specified model, the survival part of the model must be carefully assessed and the proportional hazards assumption tested. In our example, we merely assume proportionality in order to simplify the presentation. Fourthly, measurement error in the covariates and mediators can lead to biased estimates, which means that the inclusion of latent variables in this part of the model can improve the accuracy and reliability of inferences. Finally, it is important to be aware that the assumptions required in order to make causal claims based on the results of this kind of statistical model are challenging. As Judea Pearl has argued, the most important assumptions relate to the absence of confounding of each relationship that forms part of the mediation structure. In our (simple) example, we assume that there are no (significant) unmeasured common causes of affluence/deprivation, on the one hand, and (a) emergency admission to hospital, (b) hospital caseload and (c) the survival outcome, on the other.

The importance of identifying and measuring important confounders implies that a major collective effort will often be needed in order to collect and integrate the data that are required in order to draw defensible causal claims from non-experimental data. Causal models require large amounts of high-quality data, and this can necessitate costly and time-consuming data collection and data-matching techniques.

## Conclusions

The kinds of research questions that health researchers are increasingly called upon to answer are encouraging them to reconsider central aspects of their approach to theory and research practice. Above all, questions relating to mediation are provoking a rethinking of established approaches to ontology, methodology and statistics. In ontological terms, this is leading to a greater willingness to consider generative mechanisms as the object of scientific explanation. In methodological terms, it is leading to growing interest in Structural Equation Modelling as an integrated modelling framework. In statistical terms, it is focusing attention on the assumptions and conditions necessary for making causal inferences.

In this paper, we outlined the state-of-the-art in relation to mediation analysis and described the discrete-time survival model, which represents an attractive way of integrating time-to-event data and Structural Equation Modelling. We provided an example involving complex causal pathways that mediate the effects of a key socio-economic baseline covariate – social class – on the hazard of death from colon cancer following diagnosis. The results show that this approach has potential to shed light on a class of research questions which is of particular relevance in health research today.

### Statement on ethics approval

The database on which this analysis is based was provided by the National Cancer Registry Ireland. The data, once fully anonymised, are publicly available and can be requested by interested researchers. Specific ethical approval was not required for this study as the National Cancer Registry Ireland is authorised under the Health (Provision of Information) Act 1997 to collect and hold data on all persons diagnosed with cancer in the Republic of Ireland without requiring individual consent. The National Cancer Registry Ireland was established under the Health (Corporate Bodies) Act 1961 and is authorised to provide data to researchers – with due regard for anonymity – without requiring approval by an ethics committee.

## Appendix A: MPlus v5.21 code for discrete-time survival model

TITLE: Discrete-time survival model for colon cancer with proportional hazards

DATA: FILE IS G:\filename.dat;

DEFINE: IF (stage EQ 2) THEN stage2 = 1;

IF (stage NE 2) THEN stage2 = 0;

IF (stage EQ 3) THEN stage3 = 1;

IF (stage NE 3) THEN stage3 = 0;

IF (stage EQ 4) THEN stage4 = 1;

IF (stage NE 4) THEN stage4 = 0;

VARIABLE: NAMES ARE id

q1-q24 age hp2006r stage2 stage3 stage4 emerg t_col_d cl_hi_c;

USEVARIABLES = q1-q24 age hp2006r stage2 stage3 stage4 emerg t_col_d cl_hi_c;

CATEGORICAL = q1-q24;

MISSING = ALL (999);

ANALYSIS:

ESTIMATOR = MLR;

MODEL:

f BY q1-q24@1;

f@0;

f ON

age

hp2006r (dirb)

emerg (dirc)

stage2

stage3

stage4

t_col_d

cl_hi_c (dirf);

emerg ON

age

hp2006r (b1);

cl_hi_c ON

age

hp2006r (b2)

emerg (c2);

t_col_d WITH cl_hi_c;

t_col_d WITH emerg;

stage2 WITH cl_hi_c;

stage3 WITH cl_hi_c;

stage4 WITH cl_hi_c;

stage2 WITH emerg;

stage3 WITH emerg;

stage4 WITH emerg;

MODEL CONSTRAINT:

! indirect effects of DEPRIVATION

! depriv - > emerg - > F

new (indb01);

indb01 = b1*dirc;

! depriv - > caseload - > F

new (indb02);

indb02 = b2*dirf;

! depriv - > emerg - > caseload - > F

new (indb03);

indb03 = b1*c2*dirf;

! all indirect effects of deprivation

new (indb);

indb = indb01 + indb02 + indb03;

! total effect

new (totb);

totb = indb + dirb;

! mediation proportion

new (medb);

medb = indb/totb;

OUTPUT:

SAMPSTAT;

STANDARDIZED;
